# Regulation of DNA replication at the end of the mitochondrial D-loop involves the helicase TWINKLE and a conserved sequence element

**DOI:** 10.1093/nar/gkv804

**Published:** 2015-08-07

**Authors:** Elisabeth Jemt, Örjan Persson, Yonghong Shi, Majda Mehmedovic, Jay P. Uhler, Marcela Dávila López, Christoph Freyer, Claes M. Gustafsson, Tore Samuelsson, Maria Falkenberg

**Affiliations:** 1 Department of Medical Biochemistry and Cell Biology, University of Gothenburg, P.O. Box 440, SE-405 30 Gothenburg, Sweden; 2 Department of Laboratory Medicine, Karolinska Institutet, Retzius väg 8, 171 77 Stockholm, Sweden

## Abstract

The majority of mitochondrial DNA replication events are terminated prematurely. The nascent DNA remains stably associated with the template, forming a triple-stranded displacement loop (D-loop) structure. However, the function of the D-loop region of the mitochondrial genome remains poorly understood. Using a comparative genomics approach we here identify two closely related 15 nt sequence motifs of the D-loop, strongly conserved among vertebrates. One motif is at the D-loop 5′-end and is part of the conserved sequence block 1 (CSB1). The other motif, here denoted coreTAS, is at the D-loop 3′-end. Both these sequences may prevent transcription across the D-loop region, since light and heavy strand transcription is terminated at CSB1 and coreTAS, respectively. Interestingly, the replication of the nascent D-loop strand, occurring in a direction opposite to that of heavy strand transcription, is also terminated at coreTAS, suggesting that coreTAS is involved in termination of both transcription and replication. Finally, we demonstrate that the loading of the helicase TWINKLE at coreTAS is reversible, implying that this site is a crucial component of a switch between D-loop formation and full-length mitochondrial DNA replication.

## INTRODUCTION

In human cells, the mitochondrial genome (mtDNA) is a circular double-stranded molecule of 16.6 kbp (1,2). The genome encodes thirteen essential components of the oxidative phosphorylation system. Proper mtDNA replication and expression are crucial for cell viability and disturbances in these processes can cause mitochondrial diseases and have been linked to the ageing process in humans. All proteins involved in mtDNA maintenance and expression are encoded by the nuclear genome and imported into the mitochondria after protein synthesis in the cytosol. Expression of the nuclear and mtDNA genomes must therefore be coordinated in order to regulate oxidative phosphorylation capacity in response to physiological demand (3).

**Figure 1.**
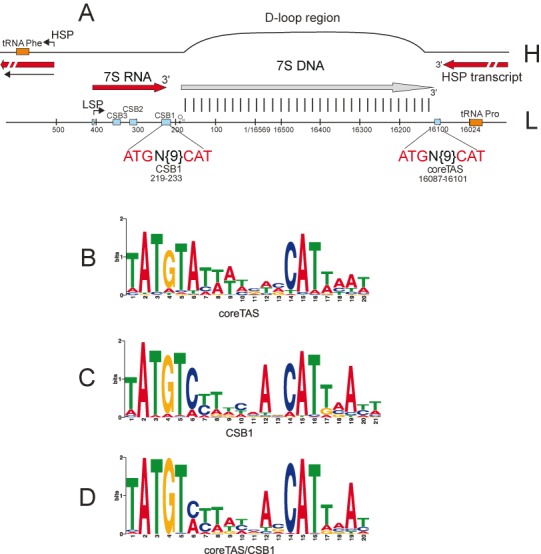
Human mitochondrial D-loop region and sequence logos of conserved motifs of ETAS1 and CSB1. (**A**) Overview of D-loop region and DNA replication and transcription termination events. Nucleotide position numbering is according to the human mitochondrial reference genome (NC_012920). Two transcripts originating from HSP and LSP, respectively, are shown (red), as well as the 7S DNA (grey). The coreTAS and CSB1 motifs are proposed to be responsible for transcription termination and in addition coreTAS might have a role in DNA replication termination, as further detailed in panels B–D and in Figures 2 and 3. (**B**) Sequence logo representing the coreTAS motif. The logo is the result of MEME analysis of vertebrate D-loop sequences, using only one strand (L-strand or forward strand in NC_012920) of the D-loop. (**C**) CSB1 sequence logo. (**D**) Represents MEME analysis as in (B and C), but motif identification was carried out by analysing both strands of the D-loop. Under these conditions the two motifs as shown in (B and C) become merged into a single motif. The logo in panel B shows the appearance on the L-strand, whereas the logos of panels (C and D) refer to the H-strand. HSP, heavy strand promoter; LSP, light strand promoter; O_H_, origin of heavy strand; CSB, conserved sequence block. The indicated O_H_ position corresponds to the major 5′-end of H-strand DNA at nucleotide position 191.

The mtDNA replication machinery contains at least five different proteins: the catalytic subunit of DNA polymerase γ (POLγA) and its processivity factor (POLγB); the replicative helicase TWINKLE; the mitochondrial single-stranded DNA-binding protein (mtSSB); and the mitochondrial RNA polymerase (POLRMT) (4). The two strands in mtDNA are designated the heavy strand (H-strand) and the light strand (L-strand), due to their strand-bias with respect to nucleotide base content. The mtDNA genome is dense in protein and RNA genes and contains only one large non-coding region of about 1000 bp (Figure 1A). This region is denoted the control region and contains the H-strand origin of replication (O_H_) as well as the promoters for transcription of the L- and H-strands (LSP and HSP, respectively) (5–8).

Mitochondrial transcription is polycistronic (9). Genome-length transcripts initiated from LSP or HSP are processed to generate mature mRNA, rRNA and tRNA molecules (6,10,11). Transcription initiated at LSP also generates RNA primers for initiation of mtDNA replication at O_H_ (12,13). The newly synthesized primer forms a stable RNA–DNA hybrid, an R-loop, near the leading-strand origin of DNA replication (14,15). The R-loop is stabilized by a G-quadruplex structure that involves both the nascent RNA and the non-template DNA strand (16,17). A polyadenylated transcript, the 7S RNA, is often discussed in terms of primer formation, but the exact function of this molecule is still not understood (10,18,19). Transcription of 7S RNA is initiated at LSP and terminates at the conserved sequence block 1 (CSB1) (3).

Biochemical and genetic evidence favour a model where mtDNA is replicated via a strand displacement mechanism with continuous DNA synthesis on both strands, although other models for mtDNA replication have been suggested (20–22). H-strand DNA replication is initiated at O_H_ and continues to displace the parental H-strand (4,22,23). During this first phase of replication, there is no simultaneous DNA synthesis on the L-strand. When H-strand replication has progressed two-thirds around the genome the replication machinery exposes the origin of L-strand DNA replication (O_L_), which adopts a stem-loop structure that function as an initiation site for L-strand DNA synthesis (12,24–26). POLRMT interacts with O_L_ and initiates transcription from a poly-dT sequence in this loop region. After about 25 nt, POLRMT is replaced by POLγ and L-strand DNA synthesis is initiated (4). After initiation at O_L_, both H- and L-strand DNA synthesis proceeds continuously until each strand is completely replicated and two daughter mtDNA molecules are formed.

More than 95% of all replication events initiated at O_H_ are prematurely terminated after about 650 nt at a series of conserved DNA sequence motifs termed termination associated sequences (TAS) (27,28). The resulting short DNA fragment is denoted 7S DNA and remains bound to the parental L-strand, while the parental H-strand is being displaced. As a result, a triple-stranded displacement loop structure, the D-loop, is formed. The function of the D-loop is still unresolved and the mechanisms that cause replication termination at TAS are unknown. However, it has been speculated that the 3′ end of the D-loop is capable of forming secondary structures, which might be involved in the termination. One possible function for the D-loop is that the pre-termination provides a switch between abortive and genome length mtDNA replication. In other words, regulation of mtDNA replication seems to take place at the level of pre-termination rather than initiation. The switch could fine-tune the mtDNA copy number in response to cellular demands (29,30).

## MATERIALS AND METHODS

### Bioinformatics

#### Mitochondrial D-loop sequences

Vertebrate mitochondrial sequences were downloaded from the NCBI. The D-loop region was extracted on the basis of annotation of the mitochondrial genome.

#### Multiple alignments of ETAS and CSB1 regions

The ETAS region of the D-loop that contains highly conserved motifs with ATG and CAT triplets was identified by using a combination of sequence similarity and profile-based searches. First, a multiple alignment was created with sequences identified with BLAST (31), FASTA (32) or SSEARCH (33,34), using the human sequence as query. The redundancy of this set of sequences was reduced by removing highly similar sequences. A profile was then produced based on the alignment and was used to search all vertebrate D-loop sequences. This profile-based step was carried out with HMMER (35). The procedure was iterated, such that the resulting sequences were again aligned, filtered for redundancy, and a profile based on the alignment was used to search vertebrate D-loop sequences. A selection of vertebrate sequences was finally aligned using ClustalW, version 2 (36).

#### MEME

The redundancy of the D-loop sequences was reduced by filtering such that any two sequences were <90% identical. A total of 1366 vertebrate sequences resulted from this filtering. Analysis with MEME (37) was then carried out using different window sizes, for instance ‘-maxsize 1 700 000 -mod zoops -dna -w 18 -nmotifs 15’ or with a similar analysis using both strands of the D-loop sequences (using parameter ‘-revcomp’). A set of 15 motifs was thus generated. The MEME analysis was carried out by either analysing only one strand of the D-loop or by analysing both strands. In the former case two related but non-identical motifs were identified. When also the complementary strand was included in the MEME analysis the CSB1 and ETAS1 motifs merged into one.

### Cell culture

HeLa-S3 cells grown in suspension were used for chromatin immunoprecipitation (ChIP), mapping of the HSP transcript, and the preparation of mitochondrial extracts. Adherent HeLa cells were used for the ddC experiments. The cells were treated with 20 µM of ddC for three days and were after that grown in medium lacking ddC (38).

### Chromatin immunoprecipitation and analysis

Mitochondria were isolated from HeLa cells as described in (39). The isolated mitochondria were cross-linked in 1% formaldehyde in phosphate buffered saline (PBS) for 10 min at room temperature. The crosslinking reaction was quenched by addition of glycine to a final concentration of 125 mM, followed by incubation for five additional minutes. Lysis, sonication and ChIP were performed as previously described (38). An aliquot of the ChIP lysate (50 μl) was taken as input control, whereas 400 μl were incubated with either 15 μl of anti-POLγA (ab2969, Abcam) or 6 μl of a homemade rabbit anti-TWINKLE antibody, or 5.5 μl of rabbit IgG (ab37415, Abcam) overnight on a rotator at 4°C. Fifty μl 50% (v/v) suspension of protein A beads (GE Healthcare) were added to the samples and incubated for 1 h at 4°C. After wash and elution, eluted DNA samples were incubated overnight at 65°C to reverse crosslinking. RNA contamination was removed by incubation with 100 ng/ml RNaseA for 15 min at 37°C. Proteins were removed by addition of 20 μg proteinase K and incubation for 2 h at 56°C. DNA was purified by phenol/chloroform extraction and ethanol precipitation. The purified DNA was used for qPCR analysis or deep-sequencing (see below). Three independent biological replicates were carried out for each ChIP reaction.

### qPCR and deep-sequencing of ChIP DNA

For qPCR analysis 2 µl of the samples were added to a reaction mix of a final concentration of 1 × iQ™ SYBR^®^ Green Supermix (Bio Rad) and 100 nM primers in a reaction of 25 µl and the DNA was amplified and quantified in a C1000™ Thermal Cycler (Bio Rad). The primer pairs corresponded to the following positions in mtDNA. Pair 1: 529–549 and 647–627; Pair 2: 369–389 and 488–468; Pair 3: 260–280 and 383–362; Pair 4: 122–141 and 240–221; Pair 5: 110–90 and 16560–11; Pair 6: 16520–16501 and 16400–16419; Pair 7: 16231–16251 and 16356–16336; Pair 8: 16054–16073 and 16186–16167; and Pair 9: 15893–15913 and 16015–15995. Quantification was performed using real time polymerase chain reaction (PCR) Software (Bio-Rad) and Excel (Microsoft). Serial dilutions of extracted input DNA (1/10 000; 1/1000; 1/100 and 1/10) were generated as a standard curve for each primer pair and for each reaction. Ratios of ChIP/input are depicted in the figures after subtracting ratios obtained from the rabbit IgG control.

For deep-sequencing of ChIP DNA, the DNA was further prepared using standard protocols provided by Illumina and deep-sequenced by using Illumina's Solexa sequencer (Beijing Genomics Institute). Quality control statistics were generated with FastQC (http://www.bioinformatics.bbsrc.ac.uk/projects/fastqc). A modified mitochondrial genome was generated for alignment of our sequencing data since the mtDNA of the HeLa cells was found to have some SNPs and Indels. Read alignments to the modified mitochondrial genome were performed using BWA (0.5.9–r16) with default parameters. The nucleotide position numbering is according to the human mitochondrial reference genome (NC_012920) although the sequence was modified to match the SNPs and indels of the HeLa cells.

### Northern blotting

Total RNA from untreated and ddC-treated HeLa cells were isolated using Trizol^®^ reagent (Invitrogen) according to the manufacturer's instructions. Purified RNA was treated with DNase I at 37°C for 30 min.

Equal amounts of total RNA were separated on a 1% formaldehyde agarose gel. The RNA was transferred onto a nylon Hybond-N+ membrane (GE Healthcare) using capillary blotting with 10× SSC buffer. After transfer of the RNA, the membrane was cross-linked by exposure to UV-light. Strand specific DNA oligonucleotides were radioactively labelled and hybridized to the membrane. Probes used for mapping of the 3′-end of the HSP transcript corresponded to the mtDNA nucleotide positions 16 009–15 986, 16 080–16 057, 16 092–16 069, 16 104–16 081, and 16116–16093 and for mapping of the 3′-end of 7S RNA the probes corresponded to the mtDNA positions 211–230, 201–220 and 191–210. Probes used for detection of the different controls were as follows; CYTB (mtDNA position 14 964–14 941) and nuclear 5.8S rRNA (5′-TCA TCG ACG CAC GAG CCG AGT GATC-3′ and 5′-CAA GTG CGT TCG AAG TGT CGA TGAT-3′).

### Southern blotting

Mitochondria from untreated and ddC-treated HeLa cells were purified by adding 0.1× of homogenization buffer (10× buffer: 400 mM Tris–HCl, pH 7.8, 250 mM NaCl, 50 mM MgCl_2_) to the cell pellet, followed by incubation for 5 min on ice. After breaking the cells with a Dounce homogenizer, homogenization buffer was added to a final concentration of 1× together with protease inhibitors. The samples were centrifuged twice at 1200 × *g* for 3 min, followed by centrifugation of the supernatant at 14 000 × *g* for 3 min. The pellets were washed in cold PBS and the samples were centrifuged again. Lysis buffer (10 mM Tris–HCl, pH 8, 100 mM NaCl, 25 mM ethylenediaminetetraacetic acid, 0.5% sodium dodecyl sulphate) and proteinase K were added to the mitochondrial pellets. After incubation for 3 h at 55°C the mtDNA was extracted with phenol/chloroform followed by ethanol precipitation. The DNA was then treated with RNase A at 37°C for 30 min, after which 800 ng of mtDNA was separated on a 1% agarose gel in 1× Tris-Borate-EDTA buffer, transferred onto a nylon Hybond-N+ membrane (GE Healthcare) and cross-linked by UV-exposure. Four radioactively 5′-end labelled strand-specific probes (mtDNA positions 16 261–16 310, 16 201–16 250, 16 441–16 490, 16 501–16 550) were hybridized to the membrane. For investigation of mouse 7S DNA extension, total DNA was isolated from mouse liver using the lysis protocol from the High Pure PCR Template Preparation Kit (Roche) followed by phenol–chloroform extraction and ethanol precipitation. Three μg DNA was separated on 1% agarose gels and transferred to a nylon Hybond-N+ as described above. Membranes were hybridized at 45°C in Rapid-hyb buffer (GE Healthcare) containing radiolabelled probes. The probe (L-strand) sequences were as follows: P1: (mtDNA positions 11–31); P2: (mtDNA positions 15 818–15 838); and P3: (mtDNA positions 15 347–15 367).

### 3′-end mapping of RNA and DNA

Total RNA was isolated from HeLa cells using TRIzol^®^ Reagent (Invitrogen). The isolated RNA was DNase I treated (Turbo DNase, Ambion) and cleaned-up with the Quick RNA kit (ZYMO research). RNA integrity was verified by agarose gel electrophoresis. RNA 3′-ends were mapped using the First Choice^®^ RLM-RACE Kit (Ambion). Briefly, polyA positive mRNA was reversely transcribed with a 3′-RACE adaptor primer generating first strand cDNA. Nested PCR, with inner (T1) and outer (T2) primers in combination with primers provided by the manufacturer, were performed for each target template. The primers used were as follows. LSP T1, 5′-TCT GGT TAG GCT GGT GTT AG-3′; LSP T2, 5′-AGA GAT GTG TTT AAG TGC TG-3′; HSP T1, 5′-ACT CCA CCA TTA GCA CCC AA-3′; and HSP T2, 5′-ATT CTC TGT TCT TTC ATG GG-3′.

For mapping of DNA 3′-ends, purified mitochondrial DNA was polyadenylated at the 3′-end using Terminal Transferase (New England Biolabs). Primers S7 T1, 5′-GTG GCT TTG GAG TTG CAG TT-3′; S7 T2, 5′-GGG TTG ATT GCT GTA CTT GC-3′ and adaptor primers (Ambion) were used to amplify the DNA fragment. PCR products were separated by agarose gel electrophoresis and cloned with the Zero blunt TOPO PCR Cloning Kit (Invitogen) before sequencing.

### Quantification of mtDNA levels

Total DNA was extracted with High Pure PCR Template Preparation Kit (Roche). The DNA samples were used for quantification of mtDNA levels. A standard curve for APP (nuclear DNA, APP-FW 5′-TTT TTG TGT GCT CTC CCA GGT CT-3′ and APP-Rev 5′-TGG TCA CTG GTT GGT TGG C-3′) and CYTB (mtDNA, CYTB-FW 5′-GCC TGC CTG ATC CTC CAA AT-3′ and CYTB-Rev 5′-AAG GTA GCG GAT GAT TCA GCC -3′) primer pairs was made with one of the DNA samples. 1 µl of the DNA samples was used for quantification of nuclear DNA whereas the samples were diluted 1:50 for the mtDNA quantification. Final concentration of the reactions were 1× iQ™ SYBR^®^ Green Supermix (Bio Rad) and 200 nM of each primer in a volume of 25 µl. A C1000™ Thermal Cycler (Bio Rad) was used.

### Rolling-circle DNA replication

The D-loop region (mtDNA position 15 914–16 569) was cloned into pBluescript SK+. Construction of the double-stranded DNA templates with a preformed replication fork was performed as described earlier (40). As a negative control, pBluescript SK+ without the D-loop region was used. Rolling-circle reactions were carried out as described previously (4) in the presence of 1 TFAM molecule per 30 bp.

### *In vitro* transcription assay

The LSP (477–398) was fused to the nucleotide positions 16 021–16 371 of human mtDNA in the direction of HSP transcription (i.e. the stronger LSP promoter replaced HSP) and cloned into pEX-A. Before transcription, the templates were linearized with XhoI, generating transcription run-off (RO) products of ∼360 nt. If the transcription terminates at coreTAS a product of about 75–90 nt should be produced instead of RO. *In vitro* transcription reactions were carried out as described previously (41) in the presence of 1 TFAM molecule per 30 bp and at a final concentration of 80 mM NaCl.

## RESULTS

### Comparative analysis of D-loop sequences reveals two related 15 nt palindromic sequence motifs that are highly conserved among vertebrates

In order to identify sequence elements in the D-loop region that are involved in the regulation of transcription and DNA replication we first used a comparative genomics approach. A schematic view of the D-loop region is shown in Figure 1A. In particular we focused our attention on the extended TAS region 1 (ETAS1) and on the site of 7S RNA transcription termination (CSB1) (42,43).

Conserved motifs were identified in available vertebrate mitochondrial D-loop sequences using two independent methods. First, a set of homologous mammalian sequences for the ETAS1 regions was identified through sequence similarity searches using the human sequence as query. We used an iterative procedure also featuring profile-based searches as described in more detail in ‘Materials and Methods’ section. This approach identified a conserved sequence of ∼20 nt and the members of this sequence family were predominantly from mammals. A sequence logo characteristic of the final set of sequences in ETAS1 is shown in Supplementary Figure S1.

We also used a second, more unbiased approach based on MEME ((37), for details see ‘Materials and Methods’ section), designed to highlight any conservation also among non-mammalian D-loop sequences. This method revealed short sequence motifs that are characteristic of the ‘central domain’ of the D-loop as well as the previously identified CSB1 (conserved sequence block), CSB2 and CSB3 sequences (Supplementary Figure S2) (42). In addition, a motif characteristic of ETAS1 (42) was identified. This motif contains strongly conserved triplets ATG and CAT, with a more variable spacer of 9 nt (Figure 1B). In the following we will refer to this motif as coreTAS. It is interesting to note that the strong conservation of the ATG and CAT triplets correlates with the lack of mutations in these positions in human individuals (Supplementary Figure S3).

We next focused our interest on CSB1 and sequence conservation of this region is further illustrated in Supplementary Figure S4. Interestingly, a MEME-based analysis revealed a motif within CSB1, which is highly reminiscent of coreTAS. Indeed, the CSB1 motif is also characterized by the triplets ATG and CAT, separated by 9 nt (compare Figure 1B and C). It should be noted that the coreTAS and CSB1 motifs are located on opposite strands (L-strand and H-strand, respectively) and on opposite sides of the D-loop region (Figure 1A). At the same time both these motifs are of a palindromic nature. The similarity between them is also illustrated by the fact that when both DNA strands (the L- and H-strands) were analysed by MEME the two motifs merged into one unified motif (Figure 1D). In the analysis of both DNA strands we also identified a less common motif with properties similar to coreTAS/CSB1, but with a spacer of 10 nt (Supplementary Figure S2).

We also noted that some vertebrate species have multiple copies of the coreTAS/CSB1 motifs (Supplementary Figure S2). Typically these are arranged in tandem. When, for instance, a motif length of 15 nt (ATG—9 nt spacer—CAT) was considered, the typical distance between two adjacent copies was 2–3 nt (data not shown). If the tandem copies of the motifs were all functional, this would suggest that the functional unit of the CSB1 and coreTAS motifs is not longer than ∼18 nt.

In conclusion, the CSB1 and coreTAS motifs are closely related and they are strongly conserved sequence elements that are found across all phyla of vertebrates, including mammals and ray-finned fishes (Supplementary Figure S4). These findings strongly suggest that the two motifs are biologically significant. On the basis of the bioinformatic analysis alone, we are not able to draw conclusions on the function of these motifs, but their palindromic nature suggests that they provide protein binding sites and that the same (or structurally related) proteins might bind to these elements.

### Mapping of the 3′-end of human 7S DNA

**Figure 2.**
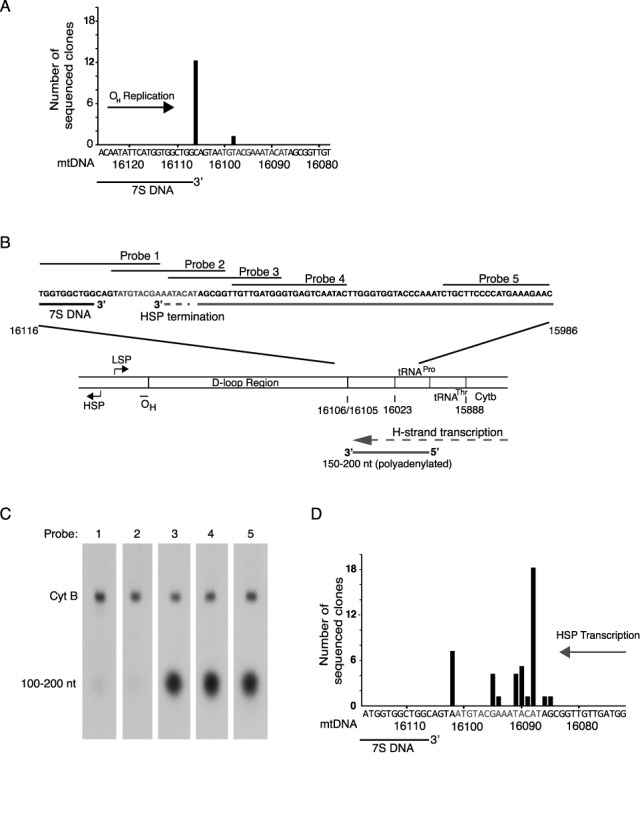
Mapping of O_H_ replication and HSP transcription termination. (**A**) Mapping of the 3′-end of 7S DNA. The coreTAS motif is shown in red. (**B**) Schematic presentation of the termination sites of 7S DNA and the HSP transcripts. The location of probes used for mapping of 3′-end of HSP transcripts are indicated as horizontal black lines. (**C**) Northern blot analysis of HSP transcripts using the probes shown in (B). (**D**) Mapping the exact 3′-end of the HSP transcripts using 3′-RACE. For abbreviations, see legend to Figure 1.

In the next set of experiments we examined the possible role of the conserved coreTAS motif. First, we mapped the 3′-end of the 7S DNA. To this end, we used a modified 3′-RACE (3′-rapid amplification of cDNA ends) protocol where a poly dA-tail was added artificially onto the 3′-ends of DNA using terminal deoxynucleotidyl transferase. We found that 7S DNA was terminated at position 16 106 (or possibly at 16 105; since the nucleotide at this position is a dA the mapping is ambiguous using this particular method), which is just 4–5 nt upstream of the coreTAS sequence (Figure 2A). The result agrees well with previous analysis of 7S DNA (28).

### Human HSP transcripts are terminated close to the 3′-end of 7S DNA

Previous mapping of HSP transcription in mouse and rat identified a transcription termination event at the area downstream of 7S DNA (44,45). We now more specifically examined if human HSP transcription is terminated at coreTAS. We designed strand-specific overlapping probes spanning the region 15 986–16 116 of mtDNA (Figure 2B) and performed northern-blotting analysis of total human RNA (Figure 2C). Our analysis identified a transcript with a 3′-end corresponding to the region close to position 16 090. This was the only transcript observed and it was detected with all three probes located outside the D-loop and the coreTAS sequence (probes 3, 4 and 5), but not with the probes closer to the D-loop (probes 1 and 2). We therefore conclude that HSP transcription terminates within the coreTAS region.

Consistent with the northern blotting data, 3′-RACE analysis showed that transcription is terminated in the coreTAS region, spanning between nucleotide positions 16 085–16 095 (Figure 2D). The major transcription termination event was located ∼20 nt downstream of the 3′-end of 7S DNA (Figure 2B). Taken together, these results suggest that coreTAS is involved in termination of both transcription and DNA replication.

### The 3′-ends of the 7S RNA transcript map to the CSB1 palindromic 15 nt region

**Figure 3.**
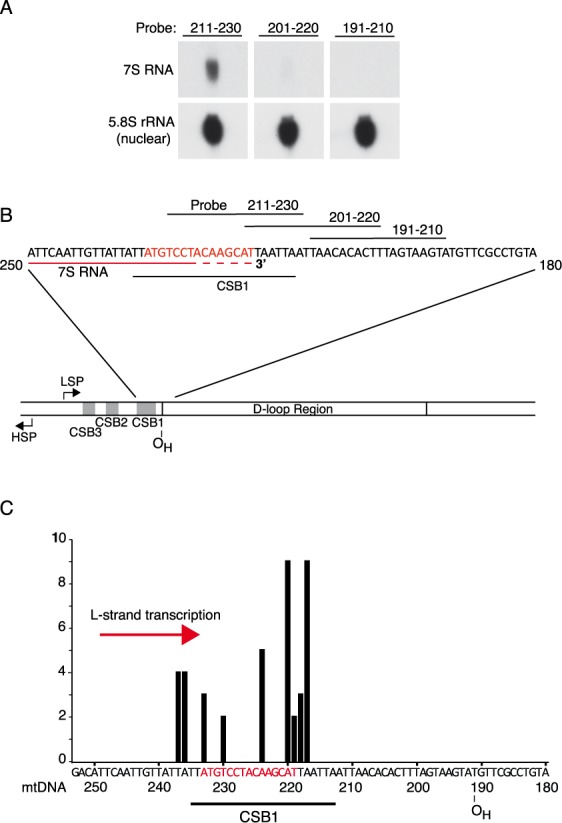
Termination of 7S RNA transcription. (**A**) Northern blot analysis of 7S RNA transcripts using the probes shown in panel B. (**B**) Schematic presentation of D-loop region with 7S RNA and CSB1. The location of probes used for mapping of the 7S RNA 3′-end is indicated as horizontal black lines. (**C**) Mapping the exact 3′-end of the 7S RNA using 3′-RACE. For abbreviations, see legend to Figure 1.

We next analysed transcription events near the CSB1 sequence element. This motif is oriented in a direction opposite to that of coreTAS. We were unable to identify any DNA ends at this site (data not shown). However, it has previously been reported that the 3′-end of a transcript referred to as 7S RNA is localized to the CSB1 region (for a review see (3)). (The reader should note that 7S RNA is not to be confused with 7S DNA, for clarification see Figure 1A). The physiological function of 7S RNA is not known although it is often discussed in the context of primer formation. Here, we mapped the 3′-end of 7S RNA using northern blotting and 3′-RACE (Figure 3A–C). The 3′-ends of the transcripts mapped to the 15 nt palindromic sequence in the CSB1 region. The pattern of the mapped 3′-ends was thus similar to that observed at coreTAS (compare Figures 2D and 3C). Our results from the 3′-end mapping of transcription termination sites are also supported by experiments carried out by Lianoglou *et al*. designed to map polyA sites in human transcriptome data from different tissues (Supplementary Figure S5) (46). We compared the 3′-ends of the transcriptome data (grey bars) with our 3′-RACE results (red bars) and found that the results for both coreTAS and CSB1 agreed well with the transcriptome data. Our observations demonstrate that both coreTAS and CSB1 coincide with transcription termination and, based on the sequence similarities between these two elements, the same mechanism might be responsible for the two termination events.

### The coreTAS sequence element cannot terminate replication/transcription on its own

One possible explanation for the transcription and DNA replication termination events is that the sequence itself has a direct role during termination (29,30). As mentioned previously, it has been suggested that the 3′-end of the D-loop is capable of forming a secondary structure, which might be involved in the termination. To address this possibility, we investigated if coreTAS could affect the progression of the mitochondrial DNA replication and transcription machineries *in vitro*. However, from these experiments, it does not seem that the coreTAS sequence itself can cause termination of replication or transcription (Supplementary Figure S6). Consistent with this conclusion, also the CSB1 sequence is by itself unable to terminate transcription (8).

### Termination of transcription and mtDNA replication at coreTAS appear to be functionally linked

Next, we examined if termination of transcription and mtDNA replication at coreTAS are functionally linked. To this end, we incubated cells with ddC, a nucleotide analogue that inhibits mtDNA synthesis and thereby leads to mtDNA depletion (47–49). It has been shown that when ddC is removed from the media, full-length mtDNA synthesis is preferred before 7S DNA synthesis (50). We wanted to investigate if reduced termination of mtDNA at coreTAS also affected termination of H-strand transcription in the opposite direction.

**Figure 4.**
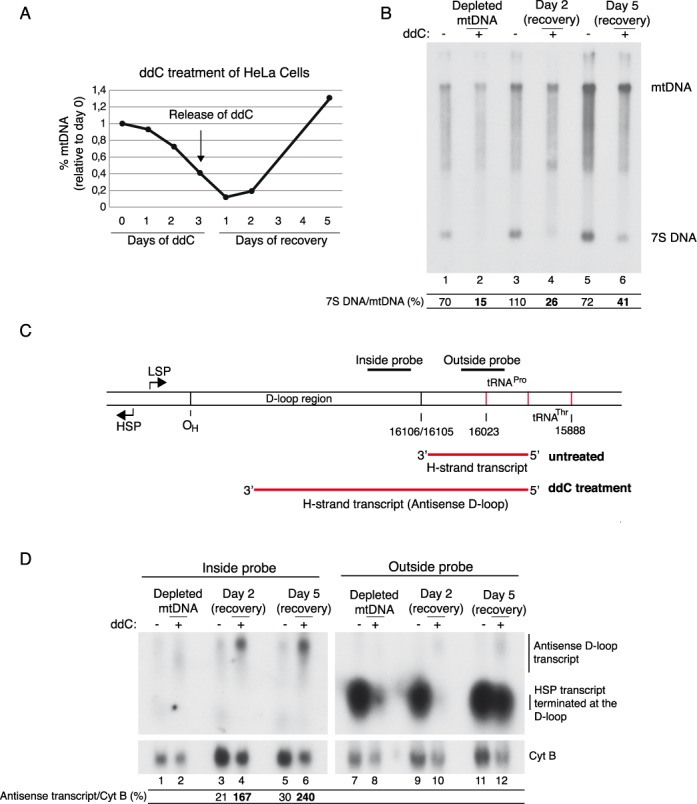
Recovery of mtDNA reduces termination of HSP transcription at coreTAS and creates longer antisense D-loop transcripts. (**A**) Quantification of mtDNA levels of ddC-treated cells. The graph shows the percentage of mtDNA levels compared to nuclear DNA levels. For details about DNA primers, see ‘Materials and Methods’ section. One representative experiment is shown. (**B**) Southern blot analysis as described in ‘Materials and Methods’ section of mtDNA from ddC treated (+) or untreated (−) cells using strand-specific probes that detect the D-loop region. The ratio of 7S DNA to mtDNA (%) is also shown. Lanes 1 and 2, untreated/ddC treated cells for 3 days; lanes 3 and 4, untreated/ddC-treated cells recovered for 2 days; and lanes 5 and 6, untreated/ddC-treated cells recovered for 5 days. (**C**) Schematic presentation of the two strand-specific probes (situated inside or outside the D-loop region) that were used for the experiment in (D). (**D**) Northern blot analysis of total RNA from ddC treated (+) or untreated (−) cells. Probes used are described in ‘Materials and Methods’ section and are depicted in (C). The levels of antisense transcript relative CYTB (%) are indicated. The experiments were repeated at least three times and one representative experiment is shown. The presented northern blots for the inside and outside probes represent two individual gels that have been run in parallel. For abbreviations, see legend to Figure 1.

As shown in Figure 4A, three days of ddC treatment led to depletion of the full-length mtDNA (to about 20%). After the ddC had been removed from the media, five days of recovery were necessary to obtain a normal level of mtDNA. Southern blotting of untreated and ddC treated cells revealed that the ratio of 7S DNA to mtDNA was changed in the ddC treated cells compared to untreated controls. Three days of ddC treatment caused a strong reduction, but did not completely abolish 7S DNA levels (Figure 4B, lane 2). After removal of ddC, we saw a gradual recovery of 7S DNA levels relative full-length mtDNA (Figure 4B). This result is in agreement with the previous finding that the premature DNA replication termination event is less frequent after mtDNA depletion (50).

Having confirmed the effect of ddC treatment on DNA replication termination, we examined if ddC treatment also affected HSP transcription termination near coreTAS. We performed northern blotting of total RNA from untreated and ddC treated cells using one probe positioned outside the D-loop and one probe positioned within the D-loop sequence (Figure 4C). In untreated cells, we detected the expected terminated short transcripts, but no defined transcript inside the D-loop region (Figure 4D, lanes 1, 3 and 5 compared to 7, 9 and 11). After 3 days treatment with ddC and after two days recovery, the terminated short transcripts are nearly abolished and we observed new longer transcripts that correspond to antisense D-loop transcripts (Figure 4D, lanes 4 and 10). These transcripts hybridized with probes both upstream and downstream of the coreTAS sequence. The longer antisense D-loop transcripts lacked a defined 3′-end and instead terminated in a broad zone inside the D-loop region (data not shown). After 5 days of recovery we could again detect the shorter transcripts but also the long antisense transcripts (Figure 4D, lanes 6 and 12). We can thus conclude that conditions that diminish termination of DNA replication at coreTAS also reduce H-strand transcription termination in the same region. It therefore appears as if these two events are functionally linked, although additional experiments are required to conclusively demonstrate this idea.

### Termination of 7S DNA replication is reversible

**Figure 5.**
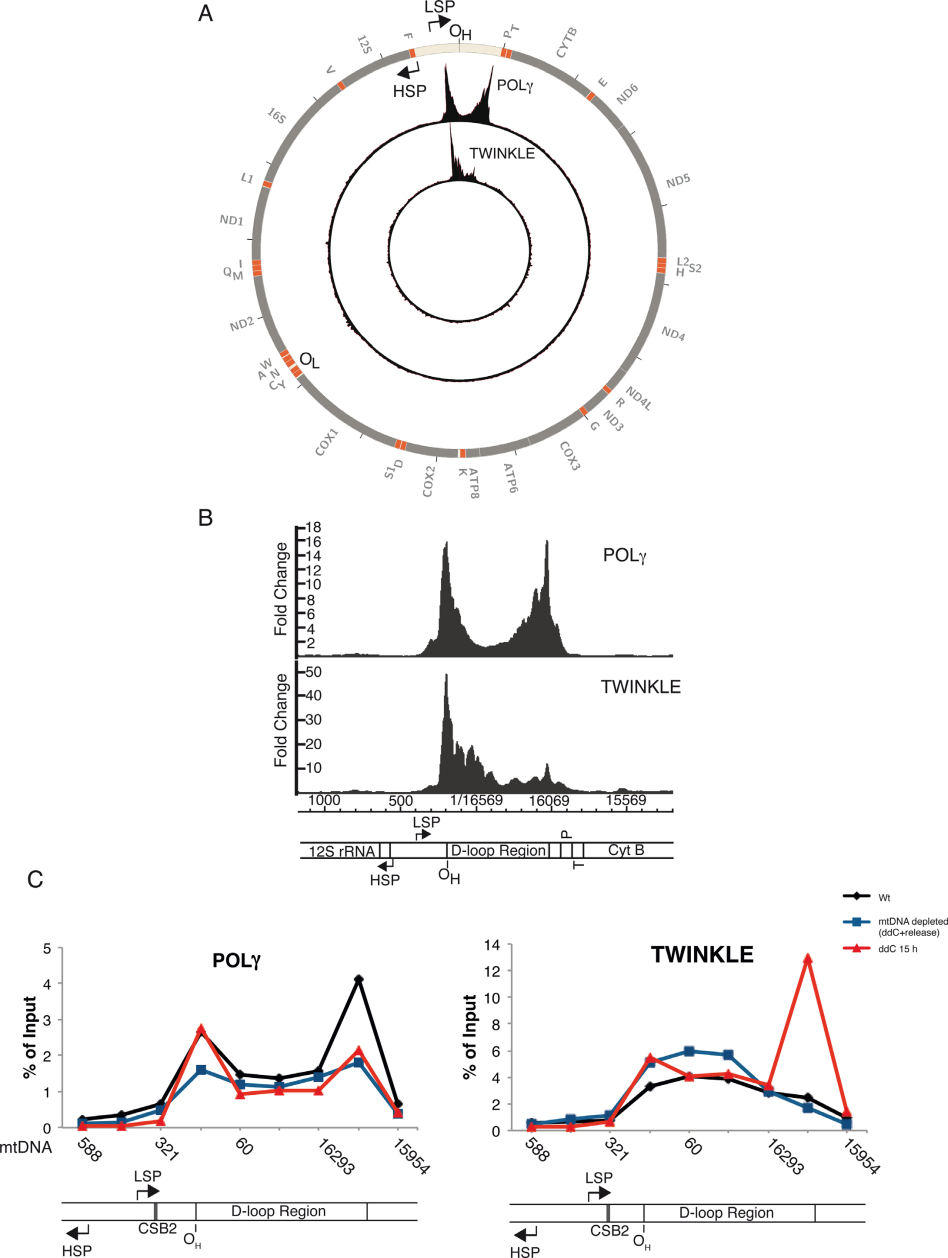
POLγ is enriched at the 3′-end of the D-loop. (**A**) Binding of POLγ and TWINKLE to the mitochondrial genome as determined using ChIP-Seq. The enrichment of POLγ and TWINKLE is indicated as fold change relative to a IgG control. (**B**) Same as in (A) but with focus on the D-loop region. (**C**) ChIP of POLγ and TWINKLE followed by quantitative PCR at the D-loop region from untreated (black, Wt) cells, ddC-treated for 3 days and then released for 3 days (blue, mtDNA depleted) or ddC-treated cells for 15 h (red). The POLγ and TWINKLE occupancies for each primer pair were calculated as percentage of input DNA using a standard curve based on serial dilutions of input DNA. One representative experiment is shown. For abbreviations, see legend to Figure 1.

A switch at the 3′-end of the D-loop seems to decide the fate of the DNA replication, i.e. if the mtDNA replication machinery should terminate or continue to synthesize full-length mtDNA. Therefore, we wanted to investigate the dynamics of the replisome at this particular site. Furthermore, we also wanted to investigate if 7S DNA can provide an alternative DNA replication initiation site for full-length mtDNA synthesis during specific metabolic conditions. We decided to examine the binding pattern of POLγ and TWINKLE before and after recovery from mtDNA depletion. First, we performed mitochondrial chromatin immunoprecipitation of POLγ and TWINKLE during normal conditions, followed by next generation sequencing (ChIP-Seq) or quantitative PCR (qPCR). The ChIP-Seq experiments revealed that both POLγ and TWINKLE were enriched within the D-loop region (Figure 5A and B). This finding is consistent with the fact that this region is replicated about 20-fold more frequently than the rest of the genome (27,28). Furthermore, the occupancy of POLγ and TWINKLE were maximal at a region overlapping with O_H_ which is consistent with previous results showing that DNA replication is initiated at O_H_ (13,24). Interestingly, a second peak of POLγ occupancy was observed around position 16 100 corresponding to the 3′-end of the 7S DNA, whereas the level of TWINKLE was relatively low at this site (Figure 5A and B). The binding patterns were also confirmed by quantitative PCR (Figure 5C, Wt). These results suggest that the termination event at the 3′-end of the D-loop is connected with TWINKLE unloading, whereas POLγ remains associated with the 3′-end of 7S DNA. In order to investigate this further we used mice expressing exonuclease-deficient POLγ (EXO-) in which a single amino acid substitution had abolished the 3′–5′ exonuclease activity (51). Wild-type POLγ lacks strand displacement activity and cannot use dsDNA as a template in the absence of TWINKLE, but impairment of the exonuclease activity allow polymerases to use dsDNA as template in the absence of a DNA helicase (52–54). If the termination and formation of 7S DNA is connected to TWINKLE unloading, EXO- POLγ should be able to extend the 7S DNA beyond the termination point independently of TWINKLE. However, if the 3′-end of 7S DNA is only a pausing site for the replisome, EXO- POLγ should not be able to extend 7S DNA. Interestingly, we found that 7S DNA is extended beyond the 3′-end of the D-loop in POLγ (EXO-) mice (Supplementary Figure S7), supporting that the termination event is connected to disassociation of the TWINKLE helicase rather than pausing of the entire replisome.

Finally, we wanted to investigate if the 7S DNA can be utilized as a primer for DNA synthesis. We therefore performed ChIP followed by qPCR using cells that had been treated with ddC for only 15 h. At this time point, 7S DNA (or mtDNA) is still not depleted and could in theory be used as a primer for DNA synthesis (Figure 4A). If 7S DNA can act as a primer, TWINKLE needs to reassemble onto the mtDNA at this location to be able to support full-length mtDNA synthesis. Interestingly, the ChIP qPCR experiment reveals that TWINKLE is highly enriched at the 3′-end of the 7S DNA rather than at the 5′-end during the conditions studied. This result is compatible with the idea that the replication machinery can restart at the 3′-end of 7S DNA when needed (Figure 5C). However, prolonged ddC treatment completely depletes 7S DNA (Figure 4B, lane 2), which in turn re-positions TWINKLE at O_H_, demonstrating that O_H_ is the primary initiation site for mtDNA replication.

**Figure 6.**
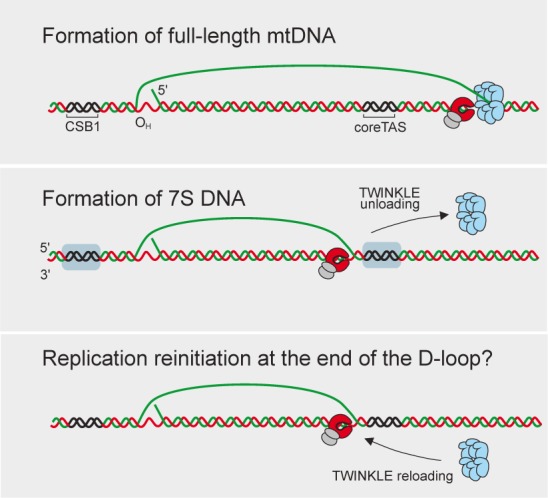
Termination of 7S DNA replication is reversible. Different scenarios of mtDNA replication are shown and molecular components include DNA polymerase (red/grey) and TWINKLE (cyan). Upper panel: replication to produce full-length mtDNA. Middle panel: replication is terminated at coreTAS and TWINKLE is unloaded. Lower panel: Model to show TWINKLE reloading and replication reinitiation at coreTAS.

In conclusion our data support a model where the termination of 7S DNA replication may be reversible. Only a small percentage of mitochondrial DNA replication events initiated at O_H_ give rise to full-length mtDNA molecules while the majority of the events are terminated prematurely at the coreTAS site. However, if needed TWINKLE can be reloaded at coreTAS and replication is continued to give rise to full-length mtDNA. This scenario is schematically depicted in Figure 6.

## DISCUSSION

Most mtDNA replication events are prematurely terminated at the 3′-end of the control region, thereby forming the D-loop. In spite of more than 40 years of research, we do not know how this termination event takes place or the functional importance of the D-loop structure (3,55). In the present report, we have investigated how the mtDNA replication machinery interacts with the D-loop region. We identified two closely related 15 nt sequence elements that are highly conserved among vertebrates and seem to be important for the dynamics of the D-loop. Our data suggest that an important function of these sequence elements is to prevent transcription to enter the D-loop region, from both 5′ and 3′ directions. One of the two palindromic sequence motifs, coreTAS, may also influence DNA replication, since 7S DNA synthesis is terminated immediately upstream of this sequence. We found that the two proteins involved in mtDNA replication, POLγ and TWINKLE, are highly enriched in the D-loop region. This enrichment is in good agreement with previous studies demonstrating that this region is replicated much more frequently (95% of all replication events are prematurely terminated) than the rest of the genome (27,28). POLγ and TWINKLE occupancy are maximal around nucleotide positions 180–200 in mtDNA, a region coinciding with O_H_. According to the strand displacement model, mtDNA replication will both initiate and terminate at this site. Together these two events could explain the observed binding of POLγ and TWINKLE at O_H_. However, the binding data do not support the strand-coupled model, which postulates that DNA synthesis is initiated in a broad zone downstream of the D-loop region in the CYTB region (56).

We noticed that the peak of POLγ and TWINKLE at O_H_ is separated from the major RNA to DNA transitions points, which are mapped about 100 nt further upstream, at CSB2. The reason for this discrepancy remains unclear. One possibility is that mtDNA synthesis is initiated at CSB2 and then paused at O_H_ before 7S DNA synthesis is continued. We know that mutations in the MGME1 nuclease lead to a shift in the 5′-end of 7S DNA towards CSB2. The observed peak of POLγ and TWINKLE may therefore be linked to MGME1-dependent processing of the initial 100 nt of synthesized DNA (57). Alternatively, the peak of POLγ and TWINKLE may not only be related to initiation of mtDNA synthesis, but also to termination and ligation, as these proteins are relocalized to the D-loop region after having replicated a full mtDNA genome. More experiments are required to address these possibilities.

In our analysis, we also found a second peak of POLγ occupancy at the 3′-end of the D-loop, suggesting that POLγ remains associated with the 3′-end of 7S DNA. The occupancy of TWINKLE at this site was relatively low which supports that this site is a replication termination site and not a pausing site. Interestingly, replication may also be reinitiated at the coreTAS region. For instance, following mild mtDNA depletion, TWINKLE levels increase in this region, suggesting that the helicase is reloaded at the 3′-end of 7S DNA and mtDNA replication is reinitiated (Figure 5C). A decrease in DNA replication termination events during mtDNA recovery is also demonstrated in the POLγ ChIP experiment, since the peak at the 3′-end of the D-loop is clearly diminished during recovery from mtDNA depletion (Figure 5C). Definitive evidence for reloading of the replication machinery at the end of the D-loop is still missing, but we hope that our findings will stimulate further research into this possibility. We favour a model in which TWINKLE is displaced by an anti-helicase activity at the 3′-end of the D-loop, but we cannot exclude the possibility that the site functions as a strong pause signal for the POLγ, leading to dissociation of TWINKLE from the replisome.

In support of this idea, we observe 3′-extended 7S DNA in the mice that are expressing an exonuclease-deficient POLγ, an enzyme which can synthesize dsDNA even in the absence of a helicase (52,53). We have also observed an activity in mitochondrial extracts that binds to the coreTAS element in gel-retardation shift experiments. However, in spite of substantial efforts, we have so far not been able to purify the DNA binding factor to homogeneity or to reveal its identity (data not shown). The activity we observe may be related to observations in bovine mitochondria, where a yet to be identified 48-kDa protein was shown to bind the D-loop region (58). A mitochondrial anti-helicase has been identified in sea urchin, where a member of the MTERF family of proteins, mtDBP, can terminate mtDNA replication (59).

An anti-helicase activity situated at the 3′-end of the D-loop may also affect H-strand transcription, since TWINKLE is a 5′–3′ helicase and thus positioned on the same strand as POLRMT during H-strand transcription (Figure 1). In support of this notion, we found that the majority of H-strand transcription is terminated at this location. This result is further supported by previous studies in human cell-lines, mouse and rat, which have identified transcription termination events in this region (42,44,60). The termination events at coreTAS may in fact be coordinately regulated, since both DNA replication and transcription termination events at this site diminish when mtDNA synthesis needs to recover after depletion. Under these conditions, H-strand transcripts continue into the D-loop region (Figure 4D) and 7S DNA synthesis is reduced in favour of full-length mtDNA synthesis.

The conserved sequence motif at CSB1 is the termination site for the 7S RNA transcript. Since the coreTAS and CSB1 motifs are closely related it appears likely that transcription termination is operating with similar mechanisms at these two sites. The function of 7S RNA is not clear but it has been suggested that it is involved in primer formation for initiation of mtDNA synthesis at O_H_ (3,61). However, 7S RNA transcripts are polyadenylated and not stably associated with the mtDNA (12,19). It could be that RNA primers are constantly produced from LSP but that the majority of these primers are not needed for DNA synthesis, and instead polyadenylated 7S RNA is produced. However, it is not obvious in such a case why transcription is terminated at CSB1 instead of producing longer LSP transcripts. One possible explanation is that transcription over the D-loop region needs to be controlled because transcription could result in inadequate 7S DNA turnover. Thus, a transcription bubble passing the triple stranded region may displace 7S DNA, leading to reannealing of the ‘parental’ H-DNA strand. Therefore, an important property of the coreTAS and CSB1 elements may be to preserve the structural integrity of the D-loop.

More work is needed to understand the function of the D-loop structure and the exact mechanism behind the DNA replication and transcription termination events that take place at coreTAS as well as CSB1. In future work, we will use functional assays to identify the missing factor that binds the conserved sequence elements and investigate its regulatory potential *in vivo*.

## Supplementary Material

SUPPLEMENTARY DATA
