# Supplementary material for: Regulation of DNA replication at the end of the mitochondrial D-loop involves the helicase TWINKLE and a conserved sequence element
Source: Nucleic Acids Res. 2015 Aug 7;43(19):9262–75. doi: 10.1093/nar/gkv804 (PMC4627069; doi:10.1093/nar/gkv804)
Supplement: SUPPLEMENTARY DATA [file supp_43_19_9262__index.html]

Regulation of DNA replication at the end of the mitochondrial D-loop involves the helicase TWINKLE and a conserved sequence element — SUPPLEMENTARY DATA 

# Regulation of DNA replication at the end of the mitochondrial D-loop involves the helicase TWINKLE and a conserved sequence element

## SUPPLEMENTARY DATA

- SUPPLEMENTARY DATA
