# Supplementary material for: Regulation of DNA replication at the end of the mitochondrial D-loop involves the helicase TWINKLE and a conserved sequence element
Source: Nucleic Acids Res. 2015 Aug 7;43(19):9262–75. doi: 10.1093/nar/gkv804 (PMC4627069; doi:10.1093/nar/gkv804)

SUPPLEMENTARY MATERIAL

Legends to Supplementary Figures

Fig. S1. Sequence logo of ETAS1 motif. Sequence logo of conserved block of ETAS1 as obtained through multiple alignment of vertebrate D-loop sequences (for details see text). Motif is shown as it appears on the L-strand.

Fig. S2. D-loop sequence conservation in vertebrates.

Sequence motif composition of selected vertebrate species are shown. Motifs were obtained using MEME and these motifs were then used by MAST to search D-loop sequences from a range of vertebrate species. CSB1-3, coreTAS and central domain motifs are highlighted. The direction shown in the same as in Fig. 1A. The p-value cutoff for including motifs in the plots was 0.01. The length of the vertical bars representing individual motifs is proportional to their p-values using a log scale (all fall in the range 1e-2 to 1e-10). Sequence logos of CSB1/coreTAS with 9 and 10 nt spacers, respectively, are shown to the upper right.

Fig. S3. Human mutations in a region containing coreTAS.

Mutations in the D-loop region of human mitochondria from MITOMAP (www.mitomap.org) are shown. Green bars show the number (1, 2 or 3) of different kinds of point mutations at the respective positions. Nucleotides in red show positions where mutations have not been identified.

Fig. S4. Alignment of D-loop region containing the CSB 1, 2 and 3 sequence elements from selected vertebrates.

Multiple alignment was constructed such that the CSB1 regions aligned according to the profile obtained by MEME. Sequences downstream of CSB1 were aligned using ClustalW, version 2, using the gapext parameter set to zero. CSB1, 2 and 3 regions are highlighted. In

addition to these conserved elements, ray-finned fishes contain the highly conserved CATAA sequence.

Fig. S5. Previously published evidence of polyA sites at coreTAS and CSB1.

Transcription termination sites inferred by analysis of data from Lianoglou et al. are shown with grey bars (46). For comparison transcription termination sites determined by 3´-RACE are shown with red bars reflecting the number of clones with a specific 3´-end position (see also Figs. 2D and 3C). Upper panel shows LSP transcription terminating at CSB1 and the lower panel shows HSP transcription termination at coreTAS (core 15 nt sequences shown in red).

Fig. S6. The sequence motif coreTAS is on its own not able to terminate replication or transcription.

A. A time course experiment using the *in vitro* rolling circle assay with two different templates (with and without the D-loop region) was performed as described in Material and Methods. The reactions were terminated at 0, 15, 30, 45, 60 and 120 minutes post-incubation. **B.** *In vitro* transcription time course experiments using a template containing the DNA sequence corresponding to the 3´-end of the D-loop (nucleotides 16021-16371) positioned in HSP direction. The reactions were terminated at 0, 5, 10, 15, 20, 25 and 30 minutes post-incubation. Run-off (RO) products ~ 360 nucleotides, expected pre-terminated products ~ 75 -90 nucleotides.

Fig. S7. The 3´- end of 7S DNA is extended by POLγ EXO- *in vivo*.

Upper panel: schematic of the D-loop and the position of oligonucleotide probes. Upstream probe (P1): - 69 nts from D-loop start; 7S DNA probe (P2); downstream probe (P3): + 32 nts from D-loop end. Middle panel: Southern blot of total liver DNA from WT or mutator mice (WT; EXO-). Lower panel: Increased exposure of the 7S DNA region.

Supplemental Fig. S1

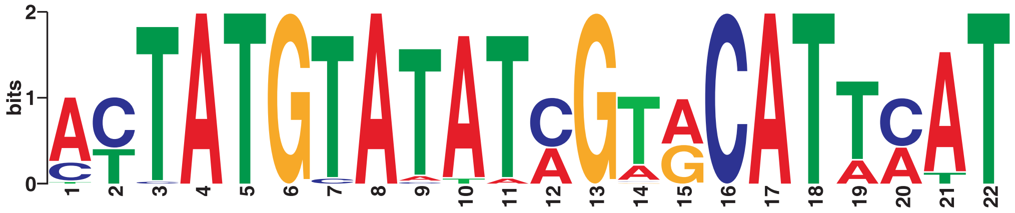

ETAS1 (L strand)

Supplemental Fig. S2

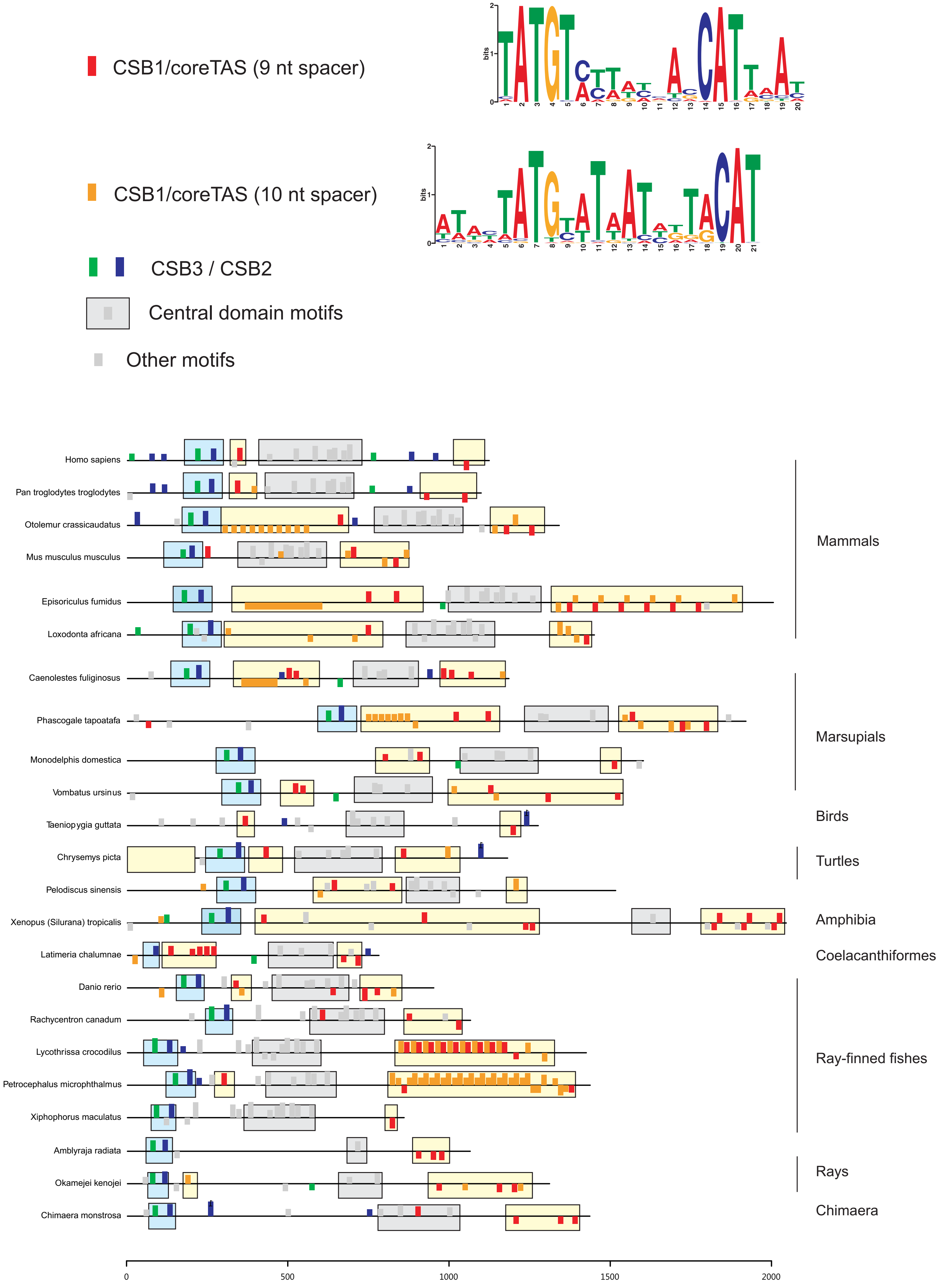

Supplemental Fig. S3

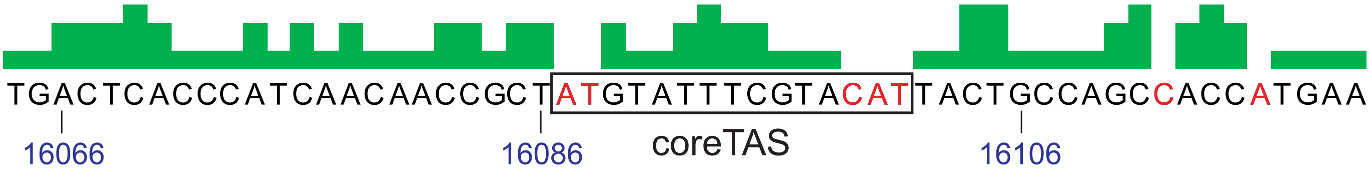

Supplemental Fig. S4

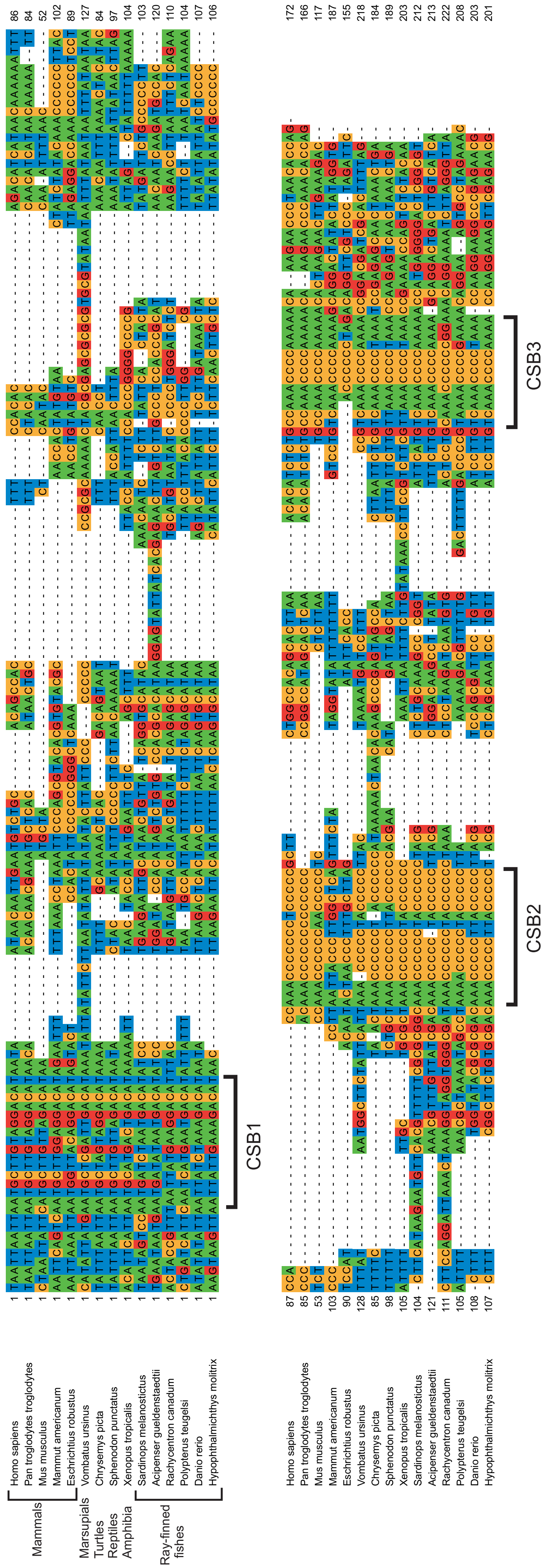

Supplemental Fig. S5

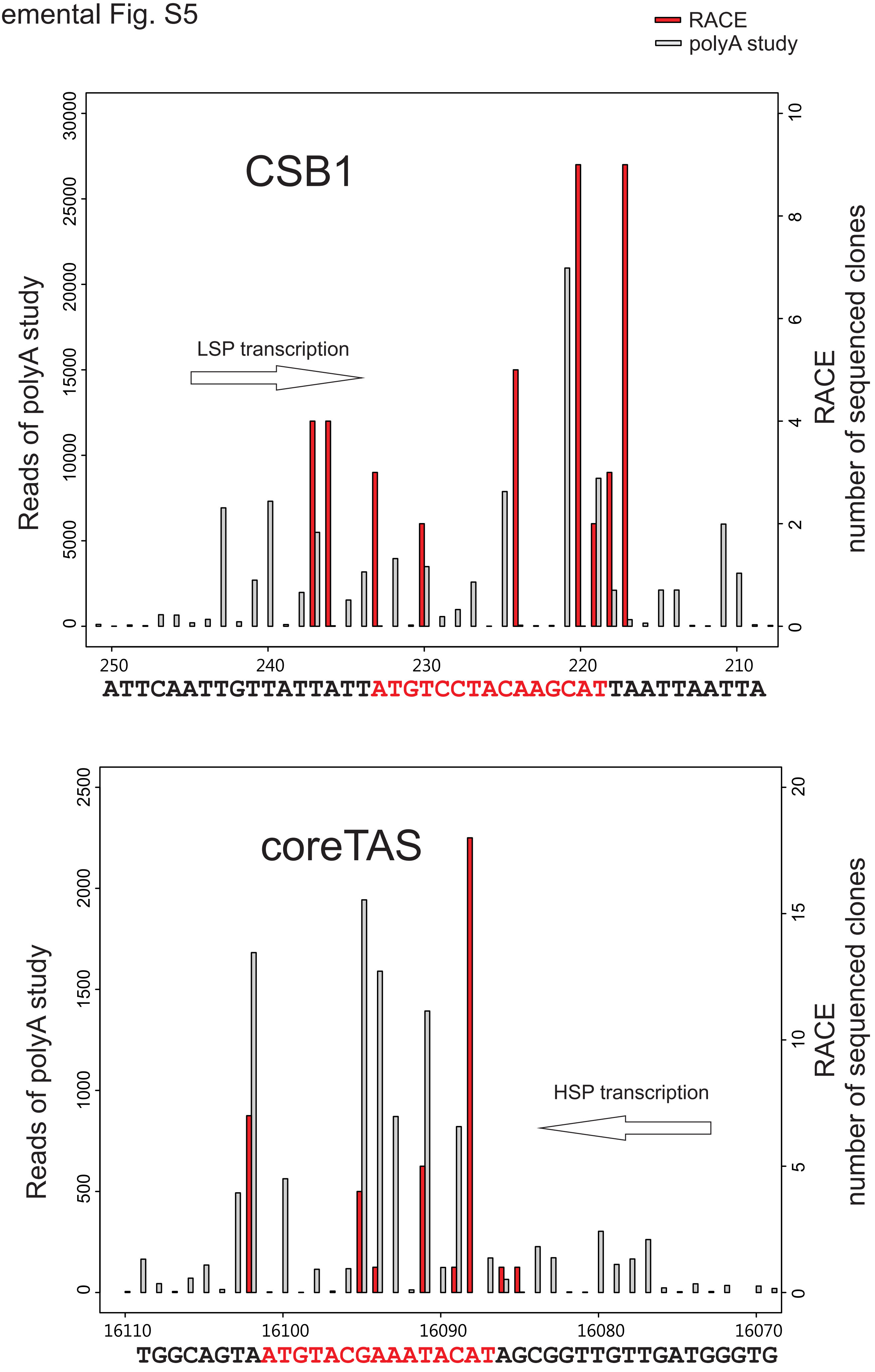

Supplementary Fig. 6 Jemt

A

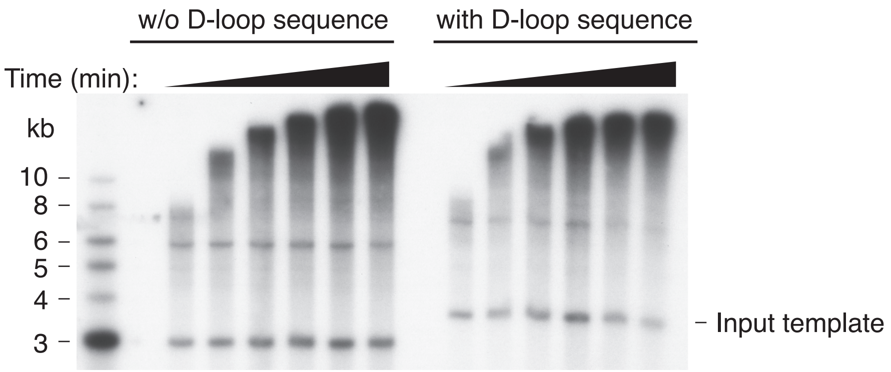

B

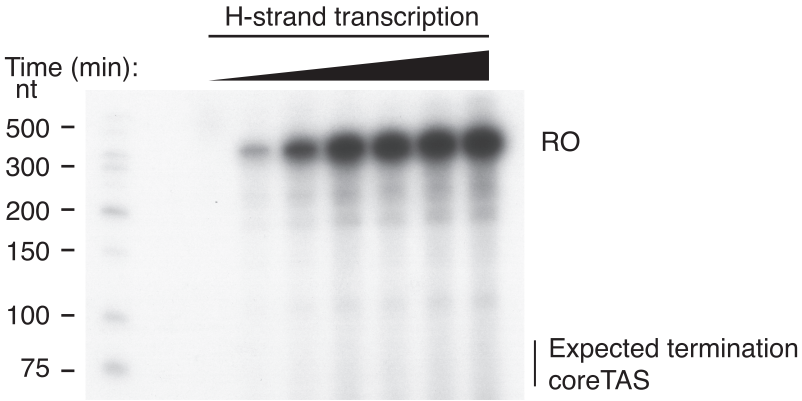

Supplementary Fig 7. Jemt

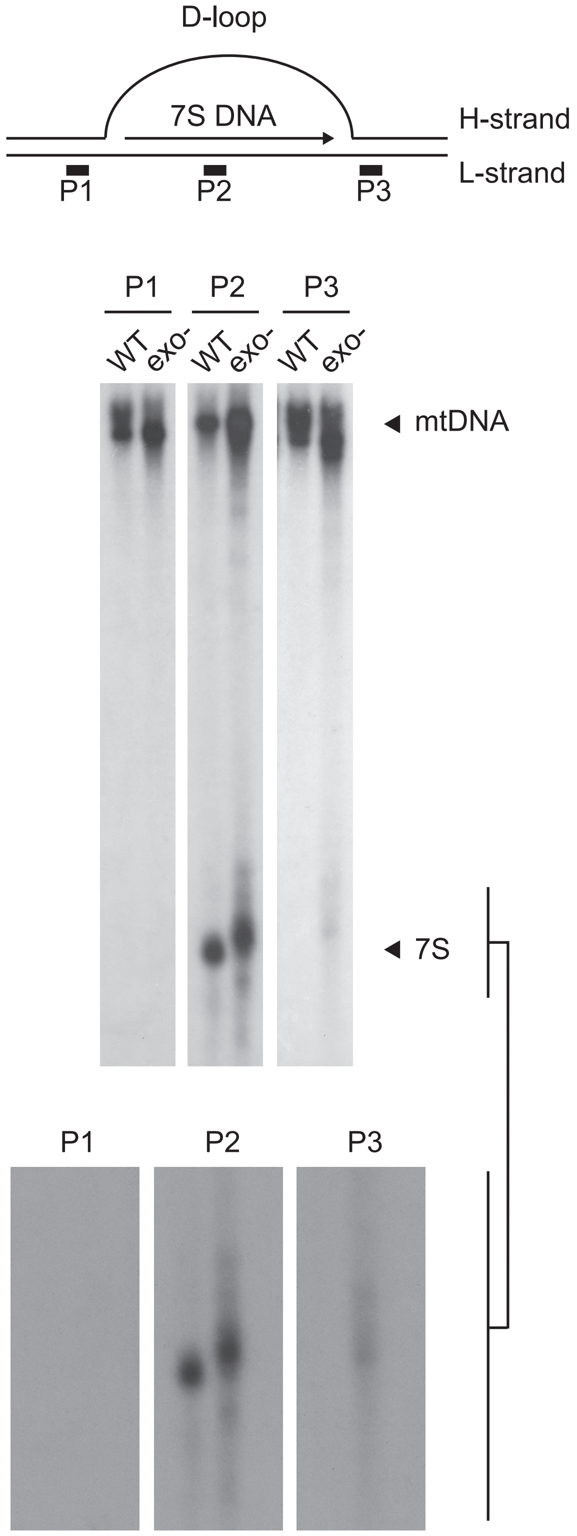

Supplement: SUPPLEMENTARY DATA [file supp_gkv804_nar-01017-y-2015-File008.pdf]
